# Report on the Sanitary Condition of the Labouring Population of Great Britain: A Supplementary Report on the Results of a Special Inquiry into the Practice of Interment in Towns

**Published:** 1844-04

**Authors:** 


### Art. VIII.

*Report on the Sanitary Condition of the Labouring Population of Great Britain: a Supplementary Report on the results of a Special Inquiry into the Practice of Interment in Towns.* By Edwin Chadwick, Esq., Barrister at Law.—*London,* 1843. 8vo, pp. 280.

We can assign two or three reasons for presenting the subject of general hygiene from time to time to our readers. The first is the conviction that the political advancement of the profession will be in proportion as medical science is applied to social economy, or in other words as it ameliorates the moral and physical condition of the people, the great object of hygiene. Secondly, to those who think less of the political aggrandisement, than of the scientific progress of the profession, we would state that the cultivation of general hygiene will necessarily lead to those innumerable observations, and the immense generalizations on which this advancement really depends, and the attainment of which has not only been beyond the reach of the profession hitherto, but will we believe remain so if no exoteric aid be afforded. A striking illustration of our argument is offered to our notice in the Report before us. Actuaries have taken it for granted, (and really upon very insufficient grounds,) that the number of persons living in each year to each death, expresses the average duration of life in years; as, for example, the deaths being at the rate of 1 in 22 annually, the average duration of life to to all is twenty-two years. Mr.

Chadwick states the proposition differently; "by nearly all statistical writers," he says, "the proportion of deaths to the population and the average ages of death are treated as equivalent," meaning however, (as appears by the context,) not the proportion of deaths to the population, but the proportion of the living to each death. However, Mr. Chadwick shows in an essay which forms an important part of the appendix attached to his 'Supplementary Report,' that this proposition is quite erroneous, and not founded on fact, and this from data obtained from the deaths in the *two millions* of the metropolitan population. Now what purely professional association, or even medical corporate body could have secured these extensive data? But Mr. Chadwick can go further; while he shows that the deaths and average ages have no relation,—in the Islington district, for example, the deaths are 1 in 55, and the average duration of life only twenty-nine years, whereas it ought theoretically to be fifty-five years, proving in fact that the average is a varying quantity,—he is enabled from the same source, to show some of the circumstances which accompany these variations, particularly those having respect to the state of the habitation, drainage, sewerage, &c., and the occupations and domestic condition of the people. We again ask what means are there *within* the profession for obtaining observations so numerous and generalizations so extensive as these? The cultivation of general hygiene, and that alone, has led, or can lead to them.

The more these matters are considered, the greater will be their acknowledged importance. Yet there is one argument more in their favour, which we cannot overlook. On a previous occasion we remarked that *the study of modern medicine is the study of human nature, and being such there are no questions concerning humanity which it may not assist to solve.* These are imperishable truths, and since the medical profession thus necessarily knows human nature better than others, that better knowledge ought to be usefully applied as well to the science of legislation and government, as to the prolongation of individual life, and the removal of individual disease.

We have made these remarks because it is but a twelvemonth since we reviewed that branch of general hygiene which refers to the disposal of the dead Those who have read Mr. Chadwick's general 'Report on the Sanitary condition of the labouring Population of Great Britain,' would expect a supplement of this kind. Like that Report it is more comprehensive and complete than any work on the subject with which we are acquainted. No available source of information has been overlooked, and we are happy to find that our own review of Riecke's publication has contributed its quota of information.

The Report commences with a discussion of the effects of putrid emanations on the public health. The negative evidence as to their innocuousness, and the facts distinctly showing their fatal effects are both scrutinized, the difficulty of effectually doing so being at the same time exhibited. The conclusion,—one which we believe none will deny,—is, that all interments in churches or in towns are injurious to the public welfare. In a second section, the harm done to the health and morals of the people by the delay of interment is exhibited. This delay is comparatively modern, since we ourselves know, from authentic documents, that at the end of the last century the corpse was rarely kept three days, in fact, was usually interred on the third day. And this delay is the more injurious, because in a large proportion of families in the metropolis (to use Mr. Chadwick's own language) one room is the bed-room, kitchen, wash-house, sitting-room, dining-room, and frequently the workroom and shop. Statistical inquiries made in the borough of Marylebone show that not more than one family in one hundred has a third room; and the same proportion was observed in the inner ward of St. George's, Hanover square. Of 1465 families residing there, 929 had only one room, and 408 not more than two; but we know, in common with many others, that there are many single rooms in the metropolis occupied by two or three families. Of course a corpse is necessarily retained in the single room when a death occurs, no matter by what disease. Mr. Chadwick gives some melancholy examples of the spread of contagious fevers from this cause. This delay of interment amongst the poor is in some degree to be attributed to their poverty, and the excessive cost of funerals. Under the latter head Mr. Chadwick gives some details exhibiting the waste and distress occasioned to the poor by the funeral societies of undertakers and publicans. At Walsall alone it is calculated that ninety societies of this kind spend £1240 per annum in eating and drinking only. A darker stain on the morals of the people is, however, shown by proved instances of labouring people entering their children in several burial societies, and then murdering them outright, or starving, or illtreating them to death to obtain the sums allowed for interment! One man got £34 3*s.* in this way, on the death of one child, from ten burial clubs.

After demonstrating the excessive cost of funerals, which in England and Wales is estimated at nearly five millions sterling, per annum, and showing that in the metropolis at least more than half of the expenditure is sheer waste, Mr. Chadwick discusses the remedies for these various evils, and at length proposes his own plan, which consists in the appointment of officers of public health. The functions of these persons would be ordinary and extraordinary. The ordinary would be the verification of the fact and cause of death by personal inspection and inquiry, and its due civic registration; the extraordinary would be the direction of such sanitary and juridical measures as the cause of death and the circumstances under which it took place might render necessary. The officer of health must combat contagion and infection by cleansing away impurities, or by the removal or embalment of the corpse. He is to detect murder and denounce it to the coroner, and assist, of course, in getting up evidence. Further: assuming the necessity of national cemeteries, he would also "have charge of the material arrangements, and take the place of the churchwardens and overseer in respect to all places of burial, and be responsible for the control of the servants of the establishment, and moreover be enabled to regulate and contract for supplies at reduced prices of materials and service of the nature of those now supplied by the undertaker." (p. 160.) The officer of health under this arrangement would also be a conductor of funerals, and indeed would be authorized to offer his services as such to surviving relatives. The salary is proposed to be fixed at 19*s.* per diem, (staff-surgeon's pay,) with an allowance for a one horse vehicle.

Mr. Chadwick works very hard himself, and plainly expects every other employée to equal him in his Herculean labours. But let our readers join us in a little contemplation of the qualifications aud duties required. First, it is absolutely necessary to the *successful* performance of his duties, that the proposed officer have a good general as well as a good medical education, and therewith a special knowledge of general hygiene and forensic medicine. He must also be of gentlemanly manners and pleasing address. Having to meet passions and prejudices, avarice and selfishness in all guises, and in all classes—he must be well acquainted alike with human nature and with business details. In short, he must be imperturbably good tempered, a man of tact, a man of business: humble, for he will have to act as an undertaker; eminently scientific and noble-minded, for he will have to apply medical science to social and political economy. Then as to the labours of the office: In the metropolis and larger towns, even the ordinary duties will be arduous and incessant. Fifteen corpses will be handled by him daily, Sundays *not* excepted, and fifteen will be daily conducted by him to the grave. Add to these the extraordinary duties: The removal of causes of disease—the investigation of suspected murders—the autopsies and analyses—and (a very important item) the return of statistical data, (Mr. Chadwick will, we are sure, provide that this duty shall not be neglected,) and we really think that our catalogue of qualifications and duties will put the staff-surgeon and his nineteen shillings *per diem* quite out of court. Indeed there are no points of comparison between the two services, except that they are both public and both medical.

Our catalogue however, is incomplete. There are factories, prisons, workhouses, lunatic-asylums, schools, manufactories, workshops, and other *quasi* public establishments to inspect; the attendance on sick prisoners, and sick poor to supervise; factory-children to look after, and adulterations and impurities in food and drink, as water, wines, butchers-meat, bread, tea, &c., to detect. These, it is true, are not mentioned by Mr. Chadwick, but they are undoubtedly an important part of the public-health officer's duties.

Mention is made of a "clerk" to the officer of health, and of a cemetery "establishment." From these and similar expressions we suspect a supplementary plan is in Mr. Chadwick's bureau. It is evident there must be a gradation of offices, and a greater complexity of difficult details than appear. The plan will be more difficult to apply to the small agricultural towns than to the metropolis and the manufacturing and commercial towns. Still more difficult will its application be to the rural manufacturing districts, such, for example, as the large parish of Rochdale, containing an area of 58,620 acres, and 84,718 inhabitants. It appears to us that the attendance on the poor, and the duties of supernumerary officer of health might, in districts like those alluded to, be combined in one individual. Of course the guardians of the poor should have no control over him. Indeed, the medical attendance on the poor is the true practical school in which the officer of health should study his duties, and should be the first grade in this new medical service. It would be his "title" to medical orders.

In an Appendix, Mr. Chadwick has supplied some interesting documents, and especially a series of most valuable statistical returns, exposing the erroneous principles in vital statistics to which we have alluded. This essay gives the *coup de grace* also to the Malthusian theory, and we cannot but congratulate Mr. Chadwick on these two important results elicited by him from the materials at his command. There is a rule of our own for correcting the errors in the averages arising from the understatement of ages which we commend to his notice, namely to the sum of the ages of all dying under one year, add the sum of the ages of as many dying under one month; and to the sum of the ages of all dying aged above one year, add the sum (corrected) of the ages of as many dying under one year. A nearly exact average is the result.

In this short notice we have by no means done justice to the valuable contents of the 'Supplementary Report.' We have discussed Mr. Chadwick's plan rather than criticised his facts, for, knowing that success is the highest praise, we are rather anxious that he should be successful in the application of "the new science of prevention" contemplated, than to give him our critical commendation. The latter is in fact unnecessary, for the volume itself speaks sufficiently for the industry, zeal, and talents of its author.

